# Real-world assessment of immunogenicity in immunocompromised individuals following SARS-CoV-2 mRNA vaccination: a two-year follow-up of the prospective clinical trial COVAXID

**DOI:** 10.1016/j.ebiom.2024.105385

**Published:** 2024-10-11

**Authors:** Puran Chen, Peter Bergman, Ola Blennow, Lotta Hansson, Stephan Mielke, Piotr Nowak, Yu Gao, Gunnar Söderdahl, Anders Österborg, C.I.Edvard Smith, Jan Vesterbacka, David Wullimann, Angelica Cuapio, Mira Akber, Gordana Bogdanovic, Sandra Muschiol, Mikael Åberg, Karin Loré, Margaret Sällberg Chen, Per Ljungman, Marcus Buggert, Soo Aleman, Hans-Gustaf Ljunggren

**Affiliations:** a Department of Medicine Huddinge, Center for Infectious Medicine, Karolinska Institutet, Stockholm, Sweden; b Department of Infectious Diseases, Karolinska University Hospital, Stockholm, Sweden; c Department of Laboratory Medicine, Clinical Immunology, Karolinska Institutet, Stockholm, Sweden; d Department of Clinical Immunology and Transfusion Medicine, Karolinska University Hospital, Stockholm, Sweden; e Department of Transplantation, Karolinska University Hospital, Stockholm, Sweden; f Department of Clinical Science, Intervention and Technology, Karolinska Institutet, Stockholm, Sweden; g Department of Hematology, Karolinska University Hospital, Stockholm, Sweden; h Department of Oncology-Pathology, Karolinska Institutet, Stockholm, Sweden; i Department of Cellular Therapy and Allogeneic Stem Cell Transplantation (CAST), Karolinska University Hospital Huddinge, Karolinska Comprehensive Cancer Center, Stockholm, Sweden; j Department of Laboratory Medicine, Biomolecular and Cellular Medicine, Karolinska Institutet, Stockholm, Sweden; k Department of Medicine Huddinge, Infectious Diseases, Karolinska Institutet, Stockholm, Sweden; l Dept of Clinical Microbiology, Karolinska University Hospital, Stockholm, Sweden; m Department of Microbiology, Tumor and Cell Biology, Karolinska Institutet, Stockholm, Sweden; n Department of Medical Sciences, Clinical Chemistry, Science for Life Laboratory, Uppsala University, Uppsala, Sweden; o Department of Medicine Solna, Karolinska Institutet, Stockholm, Sweden; p Department of Laboratory Medicine, Division of Pathology, Karolinska Institutet, Stockholm, Sweden; q Department of Medicine Huddinge, Division of Hematology, Karolinska Institutet, Stockholm, Sweden

**Keywords:** SARS-CoV-2, COVID-19, mRNA vaccine, Clinical study, Primary immunodeficiency disease, HIV, Solid organ transplantation, Hematopoietic stem cell transplantation, Chronic lymphocytic leukemia

## Abstract

**Background:**

Immunocompromised patients with primary and secondary immunodeficiencies have shown impaired responses to SARS-CoV-2 mRNA vaccines, necessitating recommendations for additional booster doses. However, longitudinal data reflecting the real-world impact of such recommendations remains limited.

**Methods:**

This study represents a two-year follow-up of the COVAXID clinical trial, where 364 of the original 539 subjects consented to participate. 355 individuals provided blood samples for evaluation of binding antibody (Ab) titers and pseudo-neutralisation capacity against both the ancestral SARS-CoV-2 strain and prevalent Omicron variants. T cell responses were assessed in a subset of these individuals. A multivariate analysis determined the correlation between Ab responses and the number of vaccine doses received, documented infection events, immunoglobulin replacement therapy (IGRT), and specific immunosuppressive drugs. The original COVAXID clinical trial was registered in EudraCT (2021-000175-37) and clinicaltrials.gov (NCT04780659).

**Findings:**

Several of the patient groups that responded poorly to the initial primary vaccine schedule and early booster doses presented with stronger immunogenicity-related responses including binding Ab titres and pseudo-neutralisation at the 18- and 24-month sampling time point. Responses correlated positively with the number of vaccine doses and infection. The vaccine response was blunted by an immunosuppressive state due to the underlying specific disease and/or to specific immunosuppressive treatment.

**Interpretation:**

The study results highlight the importance of continuous SARS-CoV-2 vaccine booster doses in building up and sustaining Ab responses in specific immunocompromised patient populations.

**Funding:**

The present studies were supported by the 10.13039/501100000781European Research Council, 10.13039/501100004047Karolinska Institutet, 10.13039/501100004063Knut and Alice Wallenberg Foundation, Nordstjernan AB, Region Stockholm, and the 10.13039/501100004359Swedish Research Council.

**Research in context**

**Evidence before this study**

Early immunogenicity-related results from the COVAXID clinical trial were reported at day 35 following two doses of anti-SARS-CoV-2 mRNA vaccine administered at days 0 and 21. Subsequently, a one-year follow-up of the COVAXID study cohort documented the dynamics of increasing binding Ab titres and pseudo-neutralisation responses following a third and fourth vaccine dose. However, several patient subgroups still responded poorly at this timepoint. These and other related findings prompted recommendations for continued booster-dose vaccinations across the studied patient groups and subgroups as well as continuous follow up studies. In this context, there remains a paucity of long-term (two years or longer) follow-up studies of SARS-CoV-2 immunogenicity-related responses in patient populations with primary and secondary immunodeficiency disorders, particularly concerning real-world outcomes derived from initially well-controlled prospective clinical trial-cohorts. In support of this notion, on July 12th, 2024, we conducted a PubMed search for “Clinical Trials” with the following search criteria: (“SARS-CoV-2” OR “COVID-19”) AND (“immunocompromised” OR “immunodeficient”) AND (“vaccination”) AND (“mRNA”), yielding eleven results. Among these, none investigated the effects of additional boosters for two years or longer. When conducted a similar PubMed search for “Clinical Studies”, the search yielded 36 results. Among these, none investigated the effects of additional boosters for two years or longer.

**Added value of this study**

This study provides longitudinal insights into immunogenicity-related responses among various immunocompromised patient groups and subgroups to SARS-CoV-2 mRNA vaccination over a two-year period, an aspect not widely covered in prior longitudinal studies. Key findings include enhanced responses in initially low-responder groups and subgroups following additional boosters, underscoring the importance of sustained vaccination efforts in these populations. Additionally, the study describes the influences of infection history, IGRT, and immunosuppressive medications, providing additional information for tailoring vaccine strategies in immunocompromised patients.

**Implications of all the available evidence**

The findings emphasize the value of personalized vaccination approaches for different groups of immunocompromised individuals. The positive association between booster doses and improved immunogenicity suggests that regular boosters are essential for building up adequate immunity in this vulnerable population. Furthermore, the interplay of infection history, IGRT, and immunosuppressive treatments indicates the need for taking these factors into consideration in managing these patients. Additional consideration should be given to the prevailing SARS-CoV-2 variants in society, and accordingly, updated vaccines towards the latter. This, and other related studies, therefore, have implications for public health policies, particularly in the context of ongoing and future vaccination strategies for COVID-19 and other related diseases.

## Introduction

In January 2020, the World Health Organization (WHO) declared Coronavirus Disease 2019 (COVID-19) a Public Health Emergency of International Concern (PHEIC), subsequently classifying it as a pandemic in March 2020.^1^^,^^2^ The pandemic has since seen the emergence of several new SARS-CoV-2 variants-of-concern (VOC).^3^^,^^4^ Early on, immunocompromised individuals were identified as high-risk groups for severe COVID-19 and death.^5^ A continuous development of strategies for COVID-19 management is needed, particularly for immunocompromised populations that may not have responded optimally to initial vaccination efforts.

Various vaccine platforms have been utilized in the development of SARS-CoV-2 vaccines,^6^ including mRNA-based vaccines.^7^ The latter demonstrated early safety and efficacy profiles in preventing severe COVID-19 and associated mortality.^8^, ^9^, ^10^ Since the pivotal mRNA vaccine trials did not include immunocompromised patient groups, there arose a need for prospective clinical trials to evaluate vaccine safety and immunological responses in these populations. As a result, the COVAXID clinical trial was initiated in early 2021 to address these concerns specifically, focusing on the anti-SARS-CoV-2 BNT162b2 mRNA vaccine in patients with primary or secondary immunodeficiencies.^11^ Initial data from this clinical trial showed that two doses of the BNT162b2 mRNA vaccine were safe, although some instances of adverse immune activation phenomena were observed. Varying degrees of Ab responses were noted two weeks following the second dose across the different immunocompromised patient groups.^11^ Subsequent one-year follow-up studies from the COVAXID clinical trial cohort revealed variable binding Ab-titres and pseudo-neutralising responses following three and four vaccine doses, with greatly diminished Omicron-specific neutralisation in several patient groups.^11^

While immunogenicity data from many studies assessing the effects of SARS-CoV-2 vaccination in immunocompromised patient groups have been reported,^12^, ^13^, ^14^, ^15^ comprehensive assessment of long-term immunogenicity-related responses in a comparative fashion including studies of both binding Ab titres and neutralisation-responses as well as cellular responses among larger sets of different immunocompromised patient groups remain limited. In this regard, the COVAXID clinical trial has offered valuable insights into immunological response development in immunocompromised patient groups following mRNA vaccination. However, questions persist regarding the long-term effects of repeated booster vaccine doses and their role in enhancing immunogenicity-relate responses in these patient groups.

This study presents comprehensive two-year follow-up data from the COVAXID cohort, evaluating the persistence and enhancement of binding Ab titres and Ab neutralization capabilities as well as cellular responses against the ancestral SARS-CoV-2 strain and emergent Omicron variants. Additionally, the study investigates the influence of additional booster doses, infection history, IGRT, and the impact of specific immunosuppressive medications on vaccine-induced responses. The study highlights benefit of repeated mRNA vaccination in immunocompromised patients and provides results of value for the formation of public health strategies and vaccination policies aimed at preventing COVID-19, particularly severe COVID-19, among these high-risk groups.

## Methods

### The COVAXID clinical trial

The prospective open-label clinical trial COVAXID (EudraCT no. 2021-000175-37) has been described.^11^ In short, inclusion criteria included individuals ≥18 years old with no known history of SARS-CoV-2 infection who had either primary or secondary immunodeficiency disorders (see below). Patients were recruited for the study during out-patient visits across various specialties at the Karolinska University Hospital, Stockholm, Sweden, with the selection process being impartial to gender. A healthy control group consisted of individuals without an immunocompromised disorder and/or immunomodulatory treatment. The original clinical trial protocol was set to conclude at 6 months after the second vaccine dose. It included two doses (days 0 and 21) of mRNA BNT162b2 (Pfizer/Biotech) and immunogenicity measurements at six timepoints (days 0, 10, 21, 35, and months 3 and 6). The clinical study was subsequently extended for a period of up to two years, with a performed analysis of results obtained at 12 months (1 year).^16^ Blood samples and associated clinical data for the two-year analysis were collected at 18 months (August 30th, 2022–November 30th, 2022) and 24 months (February 28th, 2023–May 25th, 2023). The 12 months sampling time point was median −19 days from target day (range −20.4 to −18.1 days, CI 95%), the 18 months sampling time point was median −18 days from target day (range −20.8 to −17.4 days, CI 95%) and the 24 months sampling time point was median −14 days from target day (range −16.7 to −13.4, CI 95%). 364 of the original 539 study participants consented to continue the clinical study. 355 study participants provided blood samples for analyses at 18 and/or 24 months. Of these, 334 study participants provided blood samples at month 24. Demographics data such as age and gender, and other medically relevant information were collected via electronic health records and a national vaccination register (Vaccinera), including medications, hospitalisation, as well as number and type of COVID-19 vaccinations (Table 1). Subgroups were defined based on criteria set at the initiation of the clinical trial. The average follow-up time was 721 days after the second vaccine dose (day 21). From a methodological standpoint, the blood withdrawal process and the hospital-integrated biobank workflow was standardized over the entire two-year period.

**Table 1:** Study cohort characteristics.

Study group	Subgroup defined at inclusion	Study participants (n)	Average age at inclusion (years, range)	Proportion females	Average vaccine doses (n)		Study participants w/≥1 bivalent vaccine dose (n, % of total)	Study participants on mycophenolate mofetil (MMF) (n)		Study participants on Ibrutinib (n)		Study participants on immuneglobulin replacement therapy (n)		Study participants with ≥1 confirmed SARS-CoV-2 infection (n, % of subgroup)		Hospitalized due to COVID-19 (n)	
		Entire study period	Entire study period	Entire study period	Entire study period	Month 12–24	Entire study period	Entire study period	Month 12–24	Entire study period	Month 12–24	Entire study period	Month 12–24	Entire study period	Month 12–24	Entire study period	Month 12–24
HC	>60 yrs	21	71 (62–79)	67%	5.3	1.5	8 (38, 1%)	0	0	0	0	0	0	6 (29%)	2 (10%)	0	0
	40–59 yrs	21	53 (43–59)	52%	3.3	1.0	4 (19, 0%)	0	0	0	0	0	0	10 (48%)	4 (19%)	0	0
	18–39 yrs	17	31 (22–37)	53%	3.0	1.0	2 (11, 8%)	0	0	0	0	0	0	15 (88%)	8 (47%)	0	0
	**Total**	**59**	**53 (22–79)**	**58%**	**3.9**	**1.3**	**14 (23, 7%)**	**0**	**0**	**0**	**0**	**0**	**0**	**31 (53%)**	**14 (24%)**	**0**	**0**
PID	CVID	38	53 (20–83)	58%	4.6	1.4	13 (34, 2%)	1	1	0	0	27	27	23 (61%)	13 (34%)	3	0
	XLA	2	43 (39–47)	0%	5.5	2.0	1 (50, 0%)	0	0	0	0	2	2	1 (50%)	1 (50%)	1	0
	Monogenic disease	6	36 (19–51)	67%	3.0	1.0	1 (16, 7%)	0	0	0	0	0	0	2 (33%)	0 (0%)	0	0
	CD4-cytopenia	11	55 (29–78)	73%	4.4	1.4	4 (36, 4%)	0	0	0	0	2	1	5 (45%)	2 (18%)	1	0
	Other	7	50 (22–68)	100%	4.0	1.8	4 (57, 1%)	0	0	0	0	0	0	3 (43%)	1 (14%)	0	0
	**Total**	**64**	**51 (19–83)**	**64%**	**4.3**	**1.4**	**23 (35, 9%)**	**1**	**1**	**0**	**0**	**31**	**30**	**34 (53%)**	**17 (27%)**	**5**	**0**
HIV	≤CD4 300	15	53 (24–77)	33%	3.5	1.5	5 (33, 3%)	0	0	0	0	0	0	2 (13%)	1 (7%)	0	0
	>CD4 300	40	56 (33–81)	42%	3.6	1.4	10 (25, 0%)	0	0	0	0	0	0	9 (22%)	6 (15%)	0	0
	**Total**	**55**	**55 (24–81)**	**40%**	**3.6**	**1.4**	**15 (27, 3%)**	**0**	**0**	**0**	**0**	**0**	**0**	**11 (20%)**	**7 (13%)**	**0**	**0**
HSCT	Early <6 mo	5	59 (53–68)	60%	4.2	1.7	2 (40, 0%)	0	0	0	0	2	2	3 (60%)	1 (20%)	0	0
	Intermediate 6–12 mo	10	53 (30–72)	50%	5.1	1.2	8 (80, 0%)	0	0	0	0	1	1	5 (50%)	4 (40%)	0	0
	Late >12 mo	42	61 (34–74)	50%	4.8	1.4	19 (45, 2%)	0	0	0	0	8	8	12 (29%)	5 (12%)	1	0
	CAR-T	1	55 (55–55)	0%	3.0	0.0	0 (0, 0%)	0	0	0	0	1	1	1 (100%)	1 (100%)	0	0
	**Total**	**58**	**59 (30–74)**	**50%**	**4.8**	**1.4**	**29 (50, 0%)**	**0**	**0**	**0**	**0**	**12**	**12**	**21 (36%)**	**11 (19%)**	**1**	**0**
SOT	≤6 mo w/MMF	17	51 (30–67)	29%	5.6	1.5	7 (41, 2%)	13	11	0	0	0	0	8 (47%)	3 (18%)	3	0
	>6 mo w/MMF	15	54 (31–76)	60%	6.1	1.5	6 (40, 0%)	12	11	0	0	0	0	6 (40%)	5 (33%)	1	0
	>6 mo w/o MMF	20	58 (30–79)	60%	5.3	1.2	5 (25, 0%)	0	0	0	0	0	0	12 (60%)	7 (35%)	1	0
	**Total**	**52**	**54 (30–79)**	**50%**	**5.6**	**1.4**	**18 (34, 6%)**	**25**	**22**	**0**	**0**	**0**	**0**	**26 (50%)**	**15 (29%)**	**5**	**0**
CLL	Ibrutinib	17	70 (55–87)	24%	5.8	1.8	6 (35, 3%)	0	0	13	12	6	6	8 (47%)	5 (29%)	0	0
	Off Ibrutinib	8	70 (54–86)	38%	5.6	1.3	3 (37, 5%)	0	0	3	3	3	2	3 (38%)	2 (25%)	0	0
	Indolent	25	69 (49–82)	56%	5.9	1.4	10 (40, 0%)	0	0	0	0	0	0	10 (40%)	4 (16%)	2	0
	BR/FCR	17	72 (57–84)	12%	5.8	1.4	9 (52, 9%)	0	0	1	1	6	5	9 (53%)	8 (47%)	0	0
	**Total**	**67**	**70 (49–87)**	**34%**	**5.8**	**1.5**	**28 (41, 8%)**	**0**	**0**	**17**	**16**	**15**	**13**	**30 (45%)**	**19 (28%)**	**2**	**0**

Abbreviations: HC, Healthy controls; PID, Primary immunodeficiency; HIV, Human immunodeficiency virus; HSCT, Hematopoietic stem cell transplantation; SOT, Solid organ transplantation; Chronic lymphocytic leukaemia; CVID, Common variable immune deficiency; XLA, X-linked-agammaglobulinaemia; CAR-T, Chimeric antigen receptor T-cell; MMF, Mycophenolate mofetil; BR/FCR, Bendamustine and rituximab/fludarabine, cyclophosphamide and rituximab; IGRT, Immunoglobulin replacement therapy.

Bold text represents the aggregated data for each respective study group.

### COVAXID study cohort

Patient groups with primary immunodeficiency (PID) disorders included subgroups with common variable immunodeficiency (CVID), X-linked agammaglobulinemia (XLA), monogenetic diseases, CD4-cytopenia, and other conditions. Patient groups with secondary immunodeficiency disorders included groups infected with human immunodeficiency virus (HIV) (including subgroups with ≤CD4 300 and >CD4 300 at the initiation of the clinical trial); hematopoietic stem cell transplantation (HSCT)/chimeric antigen receptor T (CAR T) cell therapy (including subgroups with HSCT within 6 months, 6–12 months, or >12 months at the initiation of the clinical trial); solid organ transplantation (SOT) (including subgroups <6 months with MMF, >6 months with MMF, and >6 months w/o MMF at the initiation of the clinical trial); and chronic lymphocytic leukaemia (CLL) (including subgroups with ibrutinib, off ibrutinib, indolent, and BR/FCR at the initiation of the clinical trial, where BR/FCR refers to “bendamustine and rituximab” and “fludarabine, cyclophosphamide and rituximab”). The control group consisted of individuals without an immunocompromised disorder or treatment, and without significant co-morbidity. The controls were selected to represent three age groups (18–39 years, 40–59 years, and >60 years at the initiation of the clinical trial). For more details, see ref.^11^

### Procedures

Adding to previous collections at days 0, 10, 21, and 35, as well as at 3, 6, 9, and 12 months, serum, plasma and PBMC for the present analyses were collected at 18 and 24 months and analysed for anti-SARS-CoV-2 Ab titres and pseudo-neutralisation as well as T cell reactivity. The study subjects received monovalent mRNA vaccines according to label during the study period (predominantly BNT162b2/Comirnaty mRNA, Pfizer-BioNTech and in some cases mRNA-1273/Spikevax, Moderna). More recently, some of the study participants obtained developed bivalent mRNA vaccines (BNT162b2 BA.1 and BNT162b2 BA.4-5, Pfizer-BioNTech) and (mRNA-1273 BA.1 and mRNA-1273 BA.4-5, Moderna) (Table 1). In rare cases (<1%), study subjects received protein vaccines (NVX-CoV2373, Novavax). Vaccine doses received by study subjects followed the recommendations of the Public Health Agency of Sweden. Clinical study-associated data were recorded in an electronic case report form (eCRF). Presence of previous SARS-CoV-2 infection was recorded via in-person or phone interviews in connection with each sampling timepoint, with grading of COVID-19 severity. Infections confirmed with PCR and/or rapid antigen test (RAT) were accepted as a verified SARS-CoV-2 infection. Conducted PCR tests were verified through a manual review of the patients' electronic health records. Home-testing (RAT) initiated at the patient's own initiative was recorded via in-person or phone interviews in connection with each sampling timepoint. Additionally, patients were retrospectively classified as having had a SARS-CoV-2 infection if IgG anti-nucleocapsid Ab titres were >5000 AU/ml (Meso Scale Discovery, MSD).

### Ab tests

Serum samples from all study subjects included were tested for IgG binding to SARS-CoV-2 Spike Wu-Hu.1 (WT, ancestral strain), and the following Omicron variants SARS-CoV-2 Spike (B.1.1.529; BA.1), SARS-CoV-2 Spike (BA.2.75), SARS-CoV-2 Spike (BA.5), SARS-CoV-2 Spike (BF.7), SARS-CoV-2 Spike (BN.1), SARS-CoV-2 Spike (BQ.1), SARS-CoV-2 Spike (BQ.1.1), SARS-CoV-2 Spike (XBB.1), SARS-CoV-2 Spike (XBB.1.5) as well as IgG binding to nucleocapsid using V-PLEX SARS-CoV-2 (Meso Scale Diagnostics, MSD). In addition, pseudo-neutralisation against SARS-CoV-2 Spike Wu-Hu.1 and the Omicron variants mentioned above was measured using V-PLEX SARS-CoV-2 (Meso Scale Diagnostics, MSD) from all study subjects included. All the above-mentioned analyses were performed at the SciLifeLab Affinity Proteomics Unit in Uppsala, Sweden. The assays were performed according to the manufacturer's instructions using a 1:50,000 dilution. Ab titres and neutralising capacity were expressed as arbitrary units (AU)/ml and % neutralisation, respectively. Additionally, serum samples were tested for pan-Ig, including IgG, to SARS-CoV-2 Wu-Hu.1 Spike receptor-binding domain (RBD) using the quantitative Elecsys anti-SARS-CoV-2 Spike enzyme immunoassay as described (Roche Diagnostics).^11^ Samples relying on the latter platform were analysed as per clinical routine, with additional dilutions if Ab titres were above the upper detection limit. Dilutions were performed to a maximum of 1:100. Since the detection of SARS-CoV-2 Abs could have been influenced by treatments with anti-SARS-CoV-2 monoclonal Abs, study subjects (n = 2) receiving long-acting anti-SARS-CoV-2 monoclonal Ab treatment were excluded from the analysis from the time point of receiving treatment.

### T cell tests

In a subgroup (n = 180) of all study subjects tested for Ab responses, PBMC were collected, and cellular samples were stimulated using peptide pools (15-mers with 11aa overlap) spanning the complete SARS-CoV-2 Spike glycoprotein (Peptide&Elephants) from ancestral Wu-Hu.1 (WT) and Omicron variant XBB.1.5. Briefly, lyophilized peptides were reconstituted at a stock concentration of 10 mg/ml in DMSO and diluted to 100 μg/ml in PBS. Cryopreserved PBMCs were thawed quickly, resuspended in complete medium in the presence of DNase I (10 U/ml; Sigma–Aldrich), and rested at 1 × 10^6^ cells/well in 96-well U-bottom plates (Corning) for 3 h at 37 °C. For surface-stained analyses, the media was then supplemented with unconjugated anti-CD40 (clone HB14; Miltenyi) followed 15 min later by the relevant peptide pool (0.5 μg/ml). Cells were then incubated at 37 °C and 5% CO_2_ for 12 h. Negative control wells contained equivalent DMSO to the peptide pool. After stimulation, cells were washed in PBS supplemented with 2% FBS and 2 mM EDTA (FACS buffer). Cells were first stained for viability at room temperature, then CCR7 at 37 °C, followed by surface markers (CD3, CD4, CD8, CD14, CD19, CD45RA, CD69, CD137 and CD154) at room temperature. Cells were fixed in Cytofix fixation buffer (BD Biosciences) and acquired using a FACSymphony A5 (BD Biosciences). Data were analysed in FlowJo (version 10). The stimulation index was calculated by dividing the frequency of the activation-induced marker (AIM) positive population divided by the equivalent population in the negative control (DMSO stimulation) sample.

### Statistical analysis

Statistical analysis was performed using Python (version 3.10.1) and the SciPy Stats library (version 1.9.2). Non-parametric tests were used for all comparative analyses due to heterogeneous study groups with small sample sizes. Bonferroni post-hoc test was used to control for type I errors. Geometric mean was used to display mean values of Ab titres. Spearman rank correlation was used for assessment of the relationship between binding Ab titres and pseudo-neutralisation responses. The statistical tests used are indicated in the figure legends. The star annotation (∗) indicates statistical significance at a p-value threshold of 0.05 (or ∗∗ for p < 0.01, ∗∗∗ for p < 0.001, ∗∗∗∗ for p < 0.0001). Additionally, non-dichotomized p-values are listed in Supplementary Table S1. For box plots, whiskers represent 1.5× IQR, with the edges of the box representing the first and third quartile. Outliers are not plotted as individual plots. Bar plots use 95% CI to display data distribution. In all figures with multiple comparisons, each comparison is indicated with a bracket with significance threshold indicated above. Non-significant differences are not displayed.

### Ethical considerations

The study was approved by the Swedish Ethical Review Board and the Swedish Medical Products Agency (no. 2021-06046-02 and no. 5.1-2021-92151, respectively). Extension of the study was approved by the same bodies. Informed consent was obtained from all study participants prior to inclusion in the study and, additionally, after the extension of the study.

### Role of funding source

The funders did not influence the study design, data collection, data analyses, interpretation, writing of the report, or decision to submit the paper for publication.

## Results

**Fig. 1:**
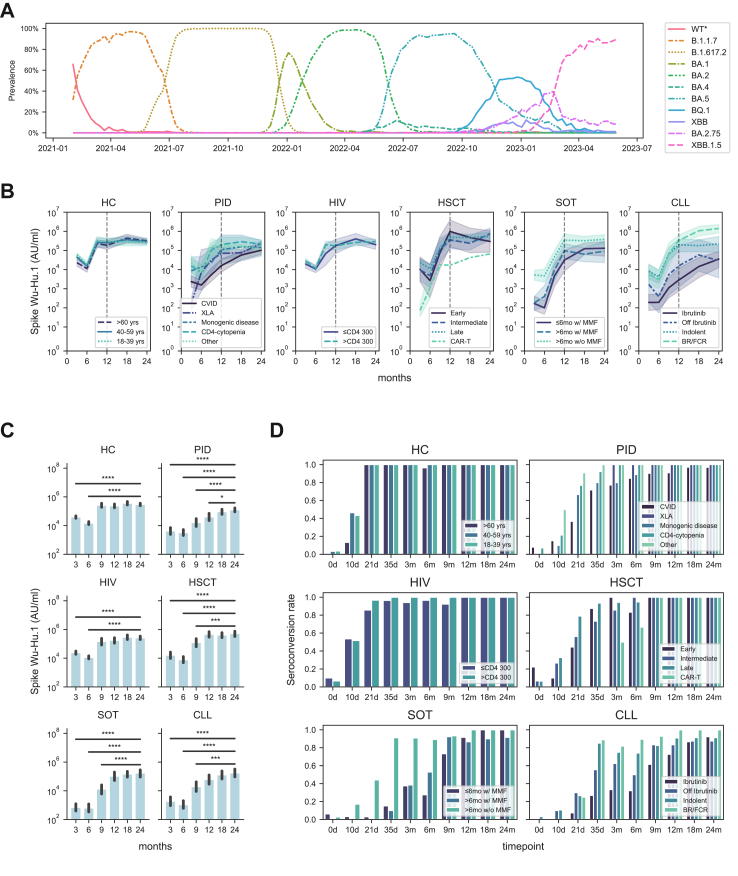
**Dynamics of antibody titres in the COVAXID cohort.** (A) Prevailing major SARS-CoV-2 subvariants in Sweden during the study period. (B) Dynamics of Spike Wu-Hu.1 Ab titres (geometric mean with 95% CI, shaded range) for each subgroup. The vertical dotted line represents the 1-year follow-up sample timepoint. (C) Bar plots showing Spike Wu-Hu.1 Ab titres at 3, 6, 9, 12, 18 and 24 months at a study group level. Statistical tests were performed using Mann–Whitney, and Bonferroni correction for multiple comparisons, using the 24-month timepoint as reference. (D) Seroconversion rates over time in each subgroup as defined by Spike receptor-binding domain (RBD) titres ≥0.8 AU/ml. The star annotation (∗) indicates statistical significance at a p-value threshold of 0.05 (or ∗∗ for p < 0.01, ∗∗∗ for p < 0.001, ∗∗∗∗ for p < 0.0001).

In a real-world setting, we assessed a total of 355 immunocompromised study participants and healthy controls (HCs) from the original COVAXID study cohort for SARS-CoV-2 immunogenicity-related responses for a period of two years. A particular emphasis was placed on the assessment of immunogenicity-related responses from year 1 (12-month sampling time point) to year 2 (24-month sampling time point with an interim analysis at 18 months). A description of the respective study groups and subgroups included in the present study is provided in Table 1. During the present 24-month period, ten different SARS-CoV-2 variants emerged which had a prevalence of over 10% at any time in Sweden (Fig. 1A).^17^ From the 12-months study point until 18 months, BA.5 and BQ.1 Omicron variants had the highest incidence. From the 18-months study point until the 24-month study point, XBB.1.5 and BA.2.75 Omicron variants had the highest incidence (Fig. 1A).

### SARS-CoV-2 binding Ab titres

First, we assessed binding Ab titres against the Wu-Hu.1 wild type (WT) SARS-CoV-2 strain over time using the V-PLEX SARS-CoV-2 platform (see Methods). Overall, two general patterns were observed within the respective study groups and subgroups (Fig. 1B and C). One pattern where study subjects had reached a plateau in terms of binding Ab titres at 12 months (or earlier) and then largely retained this level over the 18-months and 24-month sampling time points. A second pattern where study subjects had not reached a plateau at 12 months, and where binding Ab tires further increased over the 18- and the 24-month sampling time points (Fig. 1B and C). The first pattern was observed in the healthy control (HC), human immunodeficiency virus (HIV), and hematopoietic stem cell transplantation (HSCT) study groups. These groups were also among those that responded more efficiently to the first three vaccine doses.^11^ The second pattern was observed in the primary immunodeficiency (PID), solid organ transplantation (SOT), and chronic lymphocytic leukaemia (CLL) study groups. When broken down into subgroups, the PID common variable immunodeficiency (CVID) and X-linked agammaglobulinemia (XLA) subgroup as well as the CLL ibrutinib subgroup, were among those that most clearly fell into the second pattern (Supplementary Figure S1). When assessed at 24 months, the PID-CVID and CLL-ibrutinib subgroups also fell among the lowest responders in terms of SARS-CoV-2 binding Ab titres (Supplementary Figure S2A and B). Low binding Ab titres were also observed in one studied anti-CD19 CAR-T cell-treated patient (Supplementary Figure S2A and B). The results described above mimicked results obtained on the Elecsys clinical diagnostic platform (Supplementary Figure S3; compare with Fig. 1B), originally used to determine seroconversion which represented the primary endpoint of the COVAXID clinical trial (>0.8 AU/ml at day 35).^11^ With respect to seroconversion, two years after the initiation of the COVAXID clinical trial, all study subjects in the HC, HIV, and HSCT study groups had seroconverted at this or earlier time points. Notably, however, a few individual study subjects with still incomplete seroconversion at 24-months were observed in the PID-CVID subgroup, the SOT->6mo w/MMF subgroup, and in the CLL-ibrutinib, -indolent, and “-off ibrutinib” subgroups (Fig. 1D).

### SARS-CoV-2 pseudo-neutralisation responses

**Fig. 2:**
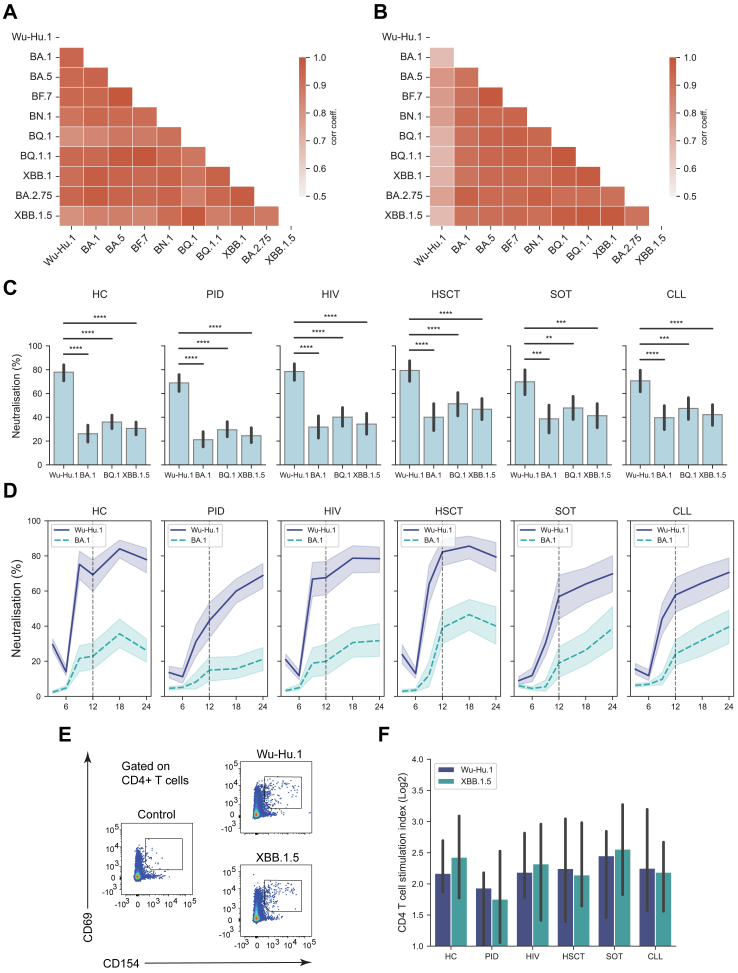
**Antibody titres and pseudo-neutralising capacity of SARS-CoV-2 subvariants.** Correlation matrices assessing (A) Ab titres and (B) pseudo-neutralising capacity of SARS-CoV-2 subvariants at the 24-month sample timepoint (Pearson correlation). (C) Bar plots showing neutralising capacity of four SARS-CoV-2 subvariants at the 24 months sampling point based on virus subvariant (upper panel) and study group (lower panel). Statistical tests were performed using Mann–Whitney, and Bonferroni correction for multiple comparisons, using healthy controls (HC) and Wu-Hu.1 (WT virus), respectively, as reference. (D) Line plot showing neutralising capacity dynamics of Spike Wu-Hu.1 and Spike BA.1 over time (geometric mean with 95% CI, shaded range). (E) Scatter plot showing correlation between binding Ab titres and pseudo-neutralising responses. Pooled data from WT and all SARS-CoV-2 variants analysed. Correlation coefficient (rho) was determined using Spearman rank correlation. Red dashed line represents locally weighted scatterplot smoothing (LOWESS). (F) Representative FACS-plot of AIM assay gated on CD4+ T cells. (G) Bar plots showing median stimulation index of CD4+ T cells against Wu-Hu.1 and XBB.1.5 calculated as a ratio between AIM + cells in peptide-stimulated wells and controls (median with 95% CI). For T cell analysis, HC (n = 23), PID (n = 27), HIV (n = 27), HSCT (n = 50), SOT (n = 22), and CLL (n = 31). The star annotation (∗) indicates statistical significance at a p-value threshold of 0.05 (or ∗∗ for p < 0.01, ∗∗∗ for p < 0.001, ∗∗∗∗ for p < 0.0001).

Earlier data from the COVAXID study group showed stable binding Ab titres across all SARS-CoV-2 variants, whereas pseudo-neutralising Ab responses were significantly impaired among Omicron subvariants compared to SARS-CoV-2 WT and non-Omicron subvariants.^16^ In the present study, we extended the above-mentioned analysis to the new Omicron variants dominating during the 12-month to 24-month period across the entire cohort. In this context, we first determined the correlation of Ab titres between WT and Omicron subvariants (including BA.1, BA.2.75, BA.5, BF.7, BN.1, BQ.1, BQ.1.1, XBB.1, XBB.1.5). Similarly to the one-year report,^16^ the newer Omicron subvariants showed a high degree of correlation with earlier Omicron variants and SARS-CoV-2 WT in terms of binding Ab titres, as assessed at the 24 months sampling time point across the entire study cohort (Fig. 2A). In a similar fashion, we also assessed pseudo-neutralising Ab responses against the WT and Omicron subvariants. Similarly to the one-year report,^16^ all Omicron subvariants showed a lower correlation with the SARS-CoV-2 WT across the entire cohort (Fig. 2B). When broken down to study groups or subgroups, pseudo-neutralisation capacity was markedly weaker across Omicron subvariants as compared to the WT strain (Fig. 2C; Supplementary Figure S4). At 24 months, the lowest Omicron pseudo-neutralising responses were observed in the PID-CVID, -monogenetic diseases, and “-other” subgroups as well as in the CLL-ibrutinib and “-off ibrutinib” subgroups (Supplementary Figure S2C and D). Similarly, low levels of pseudo-neutralisation responses were also observed in one studied anti-CD19 CAR-T cell-treated patient (Supplementary Figure S2C and D). When neutralising capacity against WT and Omicron was assessed over the study period from the three months until the 24-month sampling time point, two general patterns (like the binding Ab titres) were observed within the respective study groups (Fig. 2D). One pattern where study groups had reached a pseudo-neutralising Ab plateau at 12 months (or earlier) and then largely retained this level over the present 18 and 24-month sampling time points. A second pattern where study subjects had not reached a pseudo-neutralising Ab plateau at 12 months and where pseudo-neutralising Abs further increased over the 18- and the 24-month sampling time points (Fig. 2D). The first pattern was observed in the HC, HIV, and HSCT study groups, whereas the second pattern was observed within the PID, SOT, and CLL study groups. Of importance, at the 24-months sampling time points, the PID, SOT, and CLL study groups did not significantly differ from the HC group in terms of pseudo-neutralising responses against the WT and analysed Omicron subvariants (Fig. 2D). Finally, with the present data in hand, we determined the correlation between SARS-CoV-2 binding Ab titres and pseudo-neutralisation responses against the SARS-CoV-2 WT and all studied SARS-CoV-2 variants (Fig. 2E and Supplementary Figure S5). A generally strong correlation was observed in this respect (Spearman r >0.7).

### Cellular immune responses

Following serological analysis, we also assessed cellular immune responses against the WT and the Omicron variant XBB.1.5 at the 24-month sampling time point. Antigen-specific CD4+ T cell responses were observed across all study groups (Fig. 2F and G; Supplementary Figure S6). CD4+ T cell responses were not statistically different when patient study groups were compared to the HC group or to each other. In contrast to pseudo-neutralising Ab responses, CD4+ T cell reactivities towards WT and Omicron (XBB.1.5) antigens were strikingly similar with no statistical differences (Fig. 2E and F). Similarly, CD8+ T cell responses were also observed (Supplementary Figures S6 and S7). Nor were any of the CD8+ T cell responses statistically different when patient study groups were compared to the HC group or to each other. Furthermore, T cell responses (CD4 or CD8) were also observed among study subjects (n = 4) that had not seroconverted at the 24-month sampling time point.

### Evaluation of factors associated with Ab responses

Finally, we performed a multivariate analysis to evaluate confounding factors associated with binding Ab titres and pseudo-neutralisation in the study cohort (Table 2). Binding Ab titres and pseudo-neutralisation served as dependent variables, while the number of antigen exposures (defined as combined number of vaccine doses and infection), vaccine doses concurrent with immunosuppressive treatment, IGRT, immunosuppressive disease/condition, and age at study entry served as independent variables. Binding Ab titres and pseudo-neutralisation were evaluated in terms of responses towards WT and Omicron XBB.1.5. These responses correlated positively with the number of antigen exposures. However, the responses correlated negatively with vaccine doses concurrent with ibrutinib or MMF treatment, effectively blunting the positive effect of vaccination. Additionally, immunosuppressive states due to underlying specific diseases also negatively impacted Ab responses (Table 2). IGRT and age did not impact Ab titres or neutralisation responses. These correlations were consistent regardless of whether binding Ab titres or pseudo-neutralisation was assessed against the WT or Omicron XBB.1.5 (Table 2).

**Table 2:** Multivariate analysis of factors related to antibody titres and neutralising capacity in COVAXID study cohort.^d^

	Spike Wu-Hu.1				Spike XBB.1.5			
	coef	[0.025	0.975]	p-value	coef	[0.025	0.975]	p-value
**Binding antibody titres**								
Intercept	−51.10	−92.67	−9.53	0.016	−0.36	−1.18	0.46	0.390
# Antigen exposures^a^	17.42	10.56	24.28	<0.001	0.44	0.31	0.58	<0.001
# Vaccine doses with concurrent MMF/Ibrutinib	−14.00	−19.47	−8.52	<0.001	−0.38	−0.49	−0.27	<0.001
Immunoglobulin replacement therapy (IGRT)^c^	−18.50	−44.98	7.97	0.170	−0.15	−0.67	0.37	0.577
Immunsuppressive disease/condition^b^	−34.01	−56.12	−11.90	0.003	−1.31	−1.74	−0.87	<0.001
Age	0.56	−0.06	1.18	0.076	−0.00	−0.01	0.01	0.907
**Pseudo-neutralisation**								
Intercept	60.40	43.91	76.90	<0.001	3.93	−12.87	20.74	0.645
# Antigen exposures^a^	4.74	2.08	7.40	0.001	5.03	2.32	7.74	<0.001
# Vaccine doses with concurrent MMF/Ibrutinib	−5.76	−7.88	−3.64	<0.001	−3.84	−5.99	−1.68	0.001
Immunoglobulin replacement therapy (IGRT)^c^	1.35	−9.41	12.11	0.805	−2.26	−13.23	8.70	0.685
Immunsuppressive disease/condition^b^	−12.90	−21.40	−4.40	0.003	−12.08	−20.74	−3.42	0.006
Age	−0.12	−0.36	0.12	0.313	0.13	−0.11	0.37	0.297

a Number of vaccine doses, and RAT/PCR- and/or nucleocapsid-verified infections.

b Assigned to the subgroup CVID, XLA, Indolent, Ibrutinib or Off Ibrutinib at inclusion.

c ≥ 90 days of IGRT treatment and <90 days since last dose.

d Antibody titre measurements and pseudo-neutralisation assessments were made on the 24 month timepoint. Sex had no significant effect on the estimates.

## Discussion

In this real-world study, spanning 24 months post-start of SARS-CoV-2 mRNA vaccination, we analysed 355 of the 539 originally included study participants in the COVAXID clinical trial, including immunocompromised individuals and healthy controls, for SARS-CoV-2 immunogenicity-related responses. Analysis of anti-SARS-CoV-2 Spike Ab titres and pseudo-neutralisation responses revealed diverse patterns across the different study groups. Healthy control, HIV, and HSCT groups reached a plateau by 12 months (or earlier), while PID, SOT and CLL groups showed weaker responses at 12 months but exhibited continual increases over the entire 24-month period. At 24 months, the PID, SOT, and CLL groups had reached levels on par with the HC, HIV and HSCT groups. Correlation analysis indicated a high degree of consistency in Ab titres and pseudo-neutralising Ab responses against different Omicron variants prevailing in the population during the study period, yet pseudo-neutralising Ab responses were notably lower against Omicron variants than WT even during the 24-month sampling time point. A strong correlation was observed between SARS-CoV-2 binding Ab titres and pseudo-neutralisation responses. T cell responses were detected across all major study groups at similar levels, with reactivities being strikingly similar towards WT and Omicron XBB.1.5.

Patients with CVID or XLA typically have a low or absent capacity to generate Ab responses. It is interesting to observe how these patient subgroups non-the-less present with increasing anti-SARS-CoV-2 Ab levels over time (see e.g., Fig. 1B), in part or totally (in the latter case patients with XLA, n = 2) due to the emerging existence of anti-SARS-CoV-2 Abs in commercial IGRT products.^18^, ^19^, ^20^ However, IGRT was not associated with a significant change in anti-SARS-CoV-2 antibody titres. This could relate to different doses of IGRT provided in relation to their total IgG levels and/or the fact that these patients generally received a high number of vaccine doses potentially masking IGRT effects. Furthermore, of importance for these patient groups, vaccination contributes efficiently towards building T cell immunity (present results and^21^, ^22^, ^23^). Patients having undergone allogeneic HSCT at the onset of the present clinical trial and consequently being immunosuppressed (either myeloablative or reduced intensity, and in most cases T cell depleted) displayed remarkably high immunogenicity-related responses at the 24-month sampling time point. Several factors may contribute to this response including discontinued immune suppression, efficient immune reconstitution with time, and strong secondary immune responses following the vaccine booster doses including efficient development of memory immune responses.^24^ Earlier reports from the COVAXID cohort indicated overall similar vaccine responses in the HC and HIV, though others have noted lower responses in treated HIV groups.^25^

A striking observation during the emergence of the Omicron variant was the significant drop in virus neutralisation capacity compared to neutralisation against the WT virus.^26^^,^^27^ Notably, relatively poor neutralization responses were still observed over time towards Omicron variants even at the 24-month sampling time point in all study groups. In this respect, continued monitoring of this cohort will be of interest, especially with respect to the more recent introduction of new bivalent vaccines targeting Omicron variants.^28^^,^^29^ Notably, and in contrast to neutralisation, T cell responses at the 24 months sampling time point were strikingly similar towards WT and Omicron variants, consistent with observations from earlier time points of the present cohort.^21^^,^^30^ The similarity and robustness of T cell responses between WT and Omicron in vaccinated individuals, despite lower Ab neutralization responses against Omicron, is likely attributed to the nature of T cell antigen-recognition. T cells target multiple and broader epitopes, and hence the responses are less affected by mutation targeting specific areas of the SARS-CoV-2 Spike such as predominantly the RBD.

The findings support the need for personalized SARS-CoV-2 vaccination strategies for different groups of immunocompromised individuals. The latter could include recommendations with regards to vaccination schedules, advice for needs of regular booster doses in specific groups, needs for vaccination even in groups that cannot mount efficient Ab responses to enhance T cell responses, and recommendations of new bivalent vaccines introduced to the market. The positive correlation between booster doses and improved immunogenicity-related responses suggests that regular boosters are essential for building up adequate immunity in this vulnerable population. Additionally, an infection history contributed to built-up immunity. Thus, both booster doses and contracted infection(s) contribute(s) to increasing levels of binding Ab titres and neutralisation capacity. Immunosuppressive treatment, here illustrated by patients being on MMF (following SOT) or ibrutinib (as a treatment for CLL), clearly suppresses the ability to generate strong Ab responses following initial vaccination, but at the same time demonstrates that continuous booster doses can increase responses over time. Hence, patients within these groups likely benefit from regular revaccinations in terms of maintaining or increasing anti-SARS-CoV-2 Ab levels and neutralising capacity. In relation to measures of efficacy in relation to immunogenicity data, we did not observe any hospitalization due to severe COVID-19 in the study cohort between the 12- and 24-month sampling time point (Table 1).

The relationship between SARS-CoV-2 neutralisation levels are highly predictive of immune protection from symptomatic SARS-CoV-2 infection.^31^ However, it remains unclear whether binding and neutralization Ab titres must remain elevated (or comparable to levels in healthy individuals) in the present patient groups over time to sustain a long-term lower risk of hospitalization. This ambiguity underscores the necessity for further research to delineate the dynamics of Ab titers over time and their correlation with the risk of subsequent infection and in particular severe COVID-19 outcomes.

This study uniquely followed patients since prior to initial vaccination and onwards over two years allowing for a comprehensive assessment of immunogenicity in a real-world setting. Strengths include a well-defined patient cohort, frequent samplings, close monitoring and access to clinical data, and the ability to compare results across specific subgroups with different immunodeficiency disorders and/or conditions. Limitations include prioritization of patients for vaccine booster doses based on public health guidelines rather than a predefined clinical vaccination schedule including changes in vaccine types. Diagnosis annotation of COVID-19 relied on PCR, rapid antigen test (RAT), and/or presence of anti-nucleocapsid (NC) Ab (>5000 AU/ml), all with relative limitations regarding specificity and/or sensitivity. Notably, with respect to patients having received IGRT, we cannot exclude the possibility that some patients classified as positive using the above-mentioned criteria could be false positive due to the possible presence of NC Ab in the commercial IGRT product.

In conclusion, the present results provide a detailed assessment of immunogenicity in terms of serological responses including binding Ab titres and neutralization as well as T cell reactivities in several groups of immunocompromised patients following multiple SARS-CoV-2 mRNA vaccine booster doses, also taking the results of SARS-CoV-2 infection and immunomodulating medication into consideration. The overall results should be generalizable to similar patient groups at other sites, irrespective of sex and/or gender dimensions. The relative limit being samples are restricted to one university hospital in Sweden. Proactive measures with continuously repeated vaccinations in vulnerable patient groups, despite seroconversion and significant Ab titres against the presently dominant virus strain, may still be beneficial as it could improve cross-reactivity in case of substantial mutations in future SARS-CoV-2 variants. Importantly, repeated vaccination may also further booster T cell reactivity. Additionally, it might benefit from current updated vaccines such as those currently targeting new Omicron subvariants. In respect to this comment, we are continuing to follow this cohort in real time and aim eventually to analyze results until a final 36-month time point. The forthcoming 30 month and 36-month time-points will be of interest in context of addressing the impact of Omicron-based vaccines, not the least with respect to Omicron pseudo-neutralisation in the present patient groups.

Taken together, the present data add additional information serving to improve the management of immunocompromised patients, many of which represent risk groups for severe COVID-19. It underscores the importance of addressing known factors that interfere with vaccine responses in the individual management of vaccine regimens in immunocompromised individuals. The insights from our studies on SARS-CoV-2 mRNA vaccine responses in immunosuppressed patients should offer valuable guidance for the development and use of future mRNA vaccines targeting emerging variants and other potential viral threats in these and related patient groups.

## Contributors

PC, PB, OB, LH, SM, PN, GS, AÖ, CIES, KL, MSC, MB, PL, SA, and HGL contributed to conceptualization, funding acquisition, and discussion of data. PL and SA wrote the original clinical trial protocol. PB, LH, SM, PN, GS and SA functioned as the primary investigators for each patient study group, recruited study participants and were involved in clinical management during the initial clinical trial and the extended clinical study. OB, AÖ and JV recruited study participants and were co-responsible for clinical management during the trial. PB, LH, SM, PN, GS and SA collected and curated clinical data. PC, PB, LH, SM, PN, GS, PL, SA and HGL were responsible for project administration. PC, MA, DW, and MÅ contributed to project administration by coordinating sample collection and data collection. YG, GB, SMu and MÅ contributed to investigation through sample analyses. SA and HGL contributed to overall project administration, resources, and supervision of the trial. PC and SA have had access, and verified all underlying clinical data reported in the manuscript. PC performed and HGL contributed to the visualisation and analysis of all data. YG and OR contributed to the analysis and visualisation of T cell data. HGL performed and PC contributed to the writing of the manuscript, with final input from PB, OB, LH, SM, PN, GS, AÖ, CIES, JV, DW, AC, MÅ, KL, MSC, MB, PL, and SA. All authors have confirmed that they have had full access to the data in the study and have read and approved the manuscript prior to submission.

## Data sharing statement

Relevant data will be submitted to the European Union Drug Regulating Authorities Clinical Trials Database (EudraCT). The full original clinical study protocol is available via the SciLifeLab Data Repository (English version: https://doi.org/10.17044/scilifelab.15059364; Swedish version https://doi.org/10.17044/scilifelab.15059355). Anonymous data displayed in the manuscript will be made available upon request to the corresponding author following publication of the present article. The data, whether displayed in the manuscript or acquired during the clinical trial, will be made available in a format compliant with local regulatory authorities’ guidelines for handling patient data and in adherence to the policies of the Karolinska University Hospital and Karolinska Institutet.

## Declaration of interests

PB has received honoraria from Takeda and Novartis for educational lectures not directly relevant to this work. SM has received honoraria from Celgene/BMS, Novartis, Gilead/Kite, and DNA Prime for lectures and educational events, and as a member and/or head of data safety monitoring boards from Miltenyi and Immunicum not directly relevant to this work. CIES has received financial support from Moderna for work not directly relevant to this work. KL has received financial support from Moderna for work not directly relevant to this work. PL has received grants from Pfizer, MSD, and personal fees from Takeda, AiCuris, and OctaPharma, not directly relevant to this work. MB has served as a consultant and received honoraria from Oxford Immunotech, Gilead, MSD, BMS, Pfizer, and Mabtech, not relevant to this work. SA has received honoraria for lectures from Gilead with payment to Karolinska University Hospital and Karolinska Institutet, participated in advisory boards/consultation for Gilead and Ribocure with waived compensation not directly related to this work, and reports grants from the Swedish Research Council on COVID-19 vaccination. HGL received honoraria from Sanofi and Vycellix for consultation not relevant to this work, served on the UK-CIC Oversight Committee, led the Karolinska Institutet COVID-19 vaccine group, and is on the scientific advisory group for the International Vaccine Institute. All other authors declare no potential or actual conflict of interest to the work presented in this paper.

## Appendix A Supplementary data

SupplementalDataBinder
