# Supplementary material for: Real-world assessment of immunogenicity in immunocompromised individuals following SARS-CoV-2 mRNA vaccination: a two-year follow-up of the prospective clinical trial COVAXID
Source: eBioMedicine. 2024 Oct 11;109:105385. doi: 10.1016/j.ebiom.2024.105385 (PMC11663764; doi:10.1016/j.ebiom.2024.105385)
Supplement: SupplementalDataBinder [file mmc1.pdf]

Supplementary Figure S1

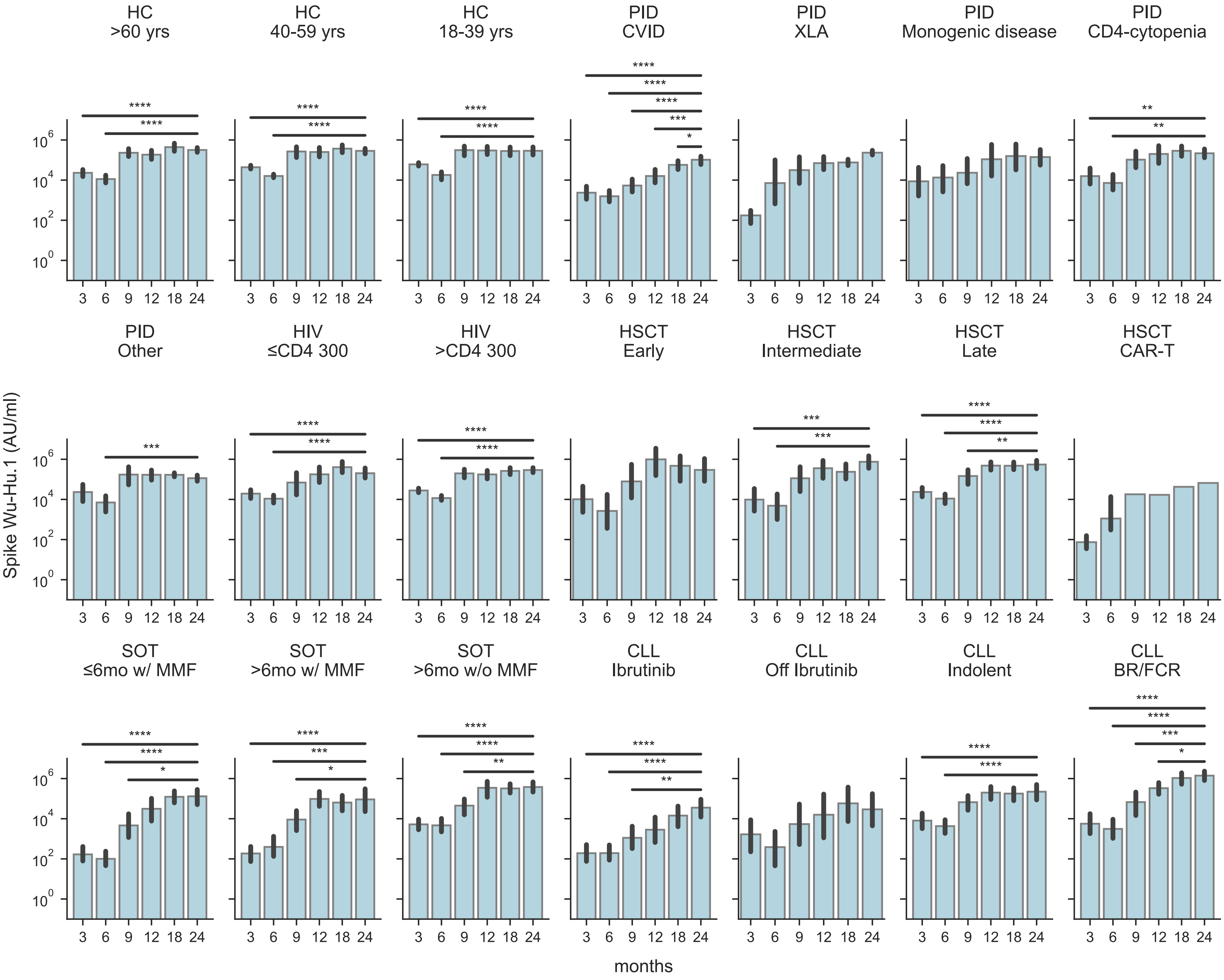

Supplementary Figure S2

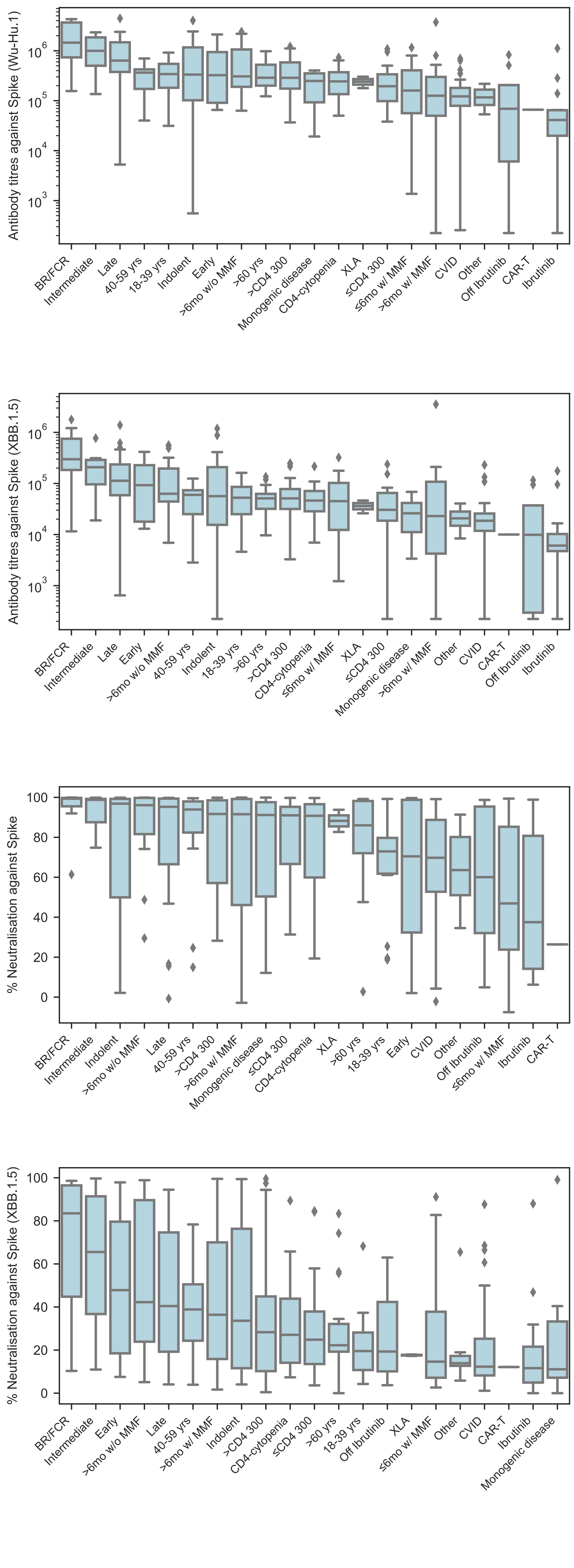

Supplementary Figure S3

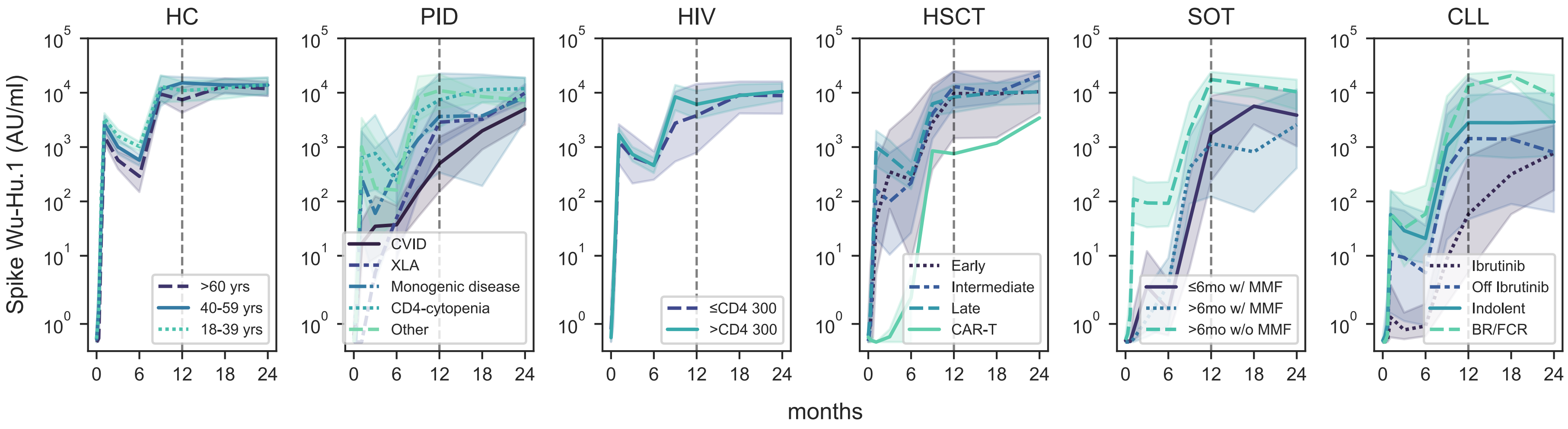

Supplementary Figure S4

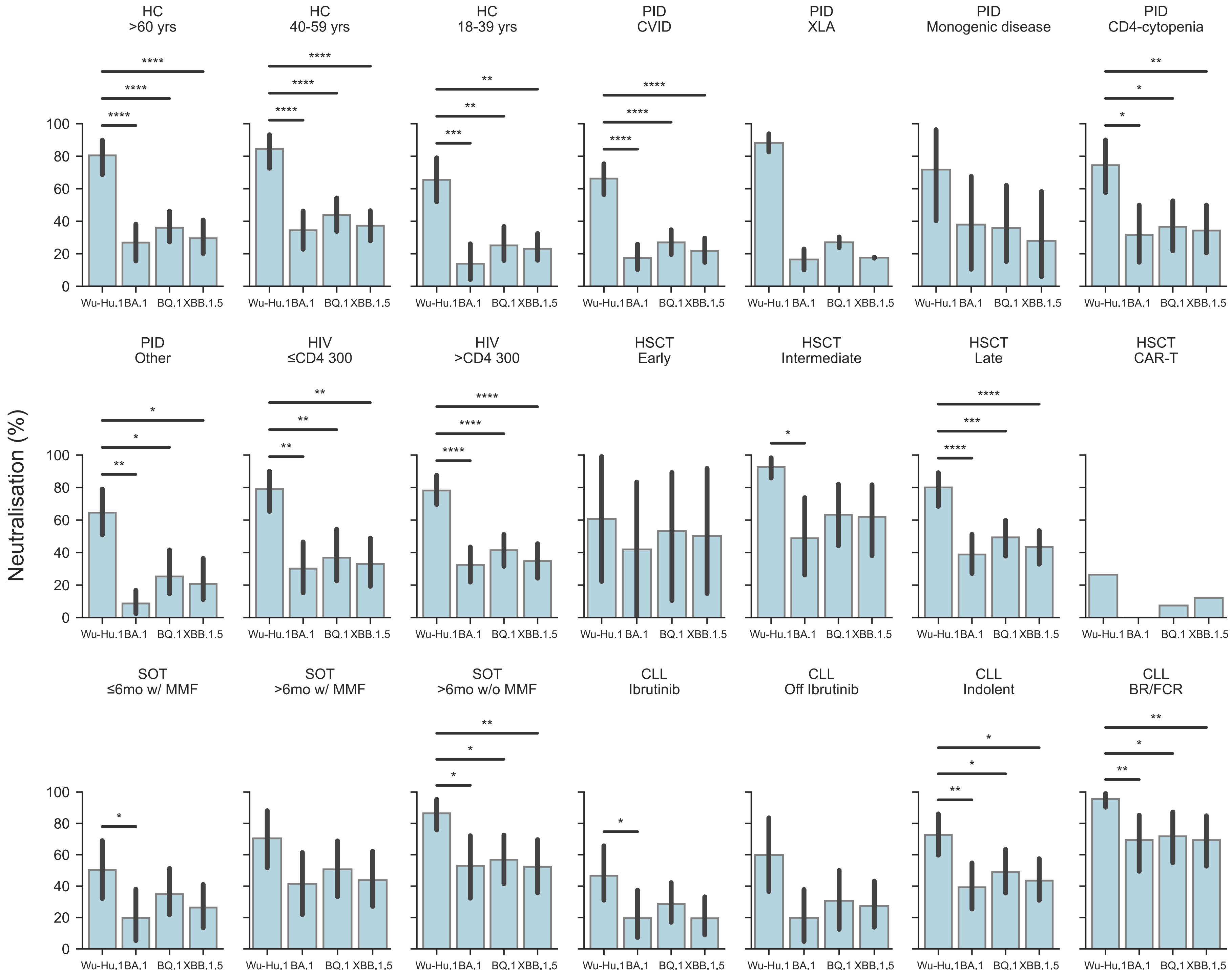

Supplementary Figure S5

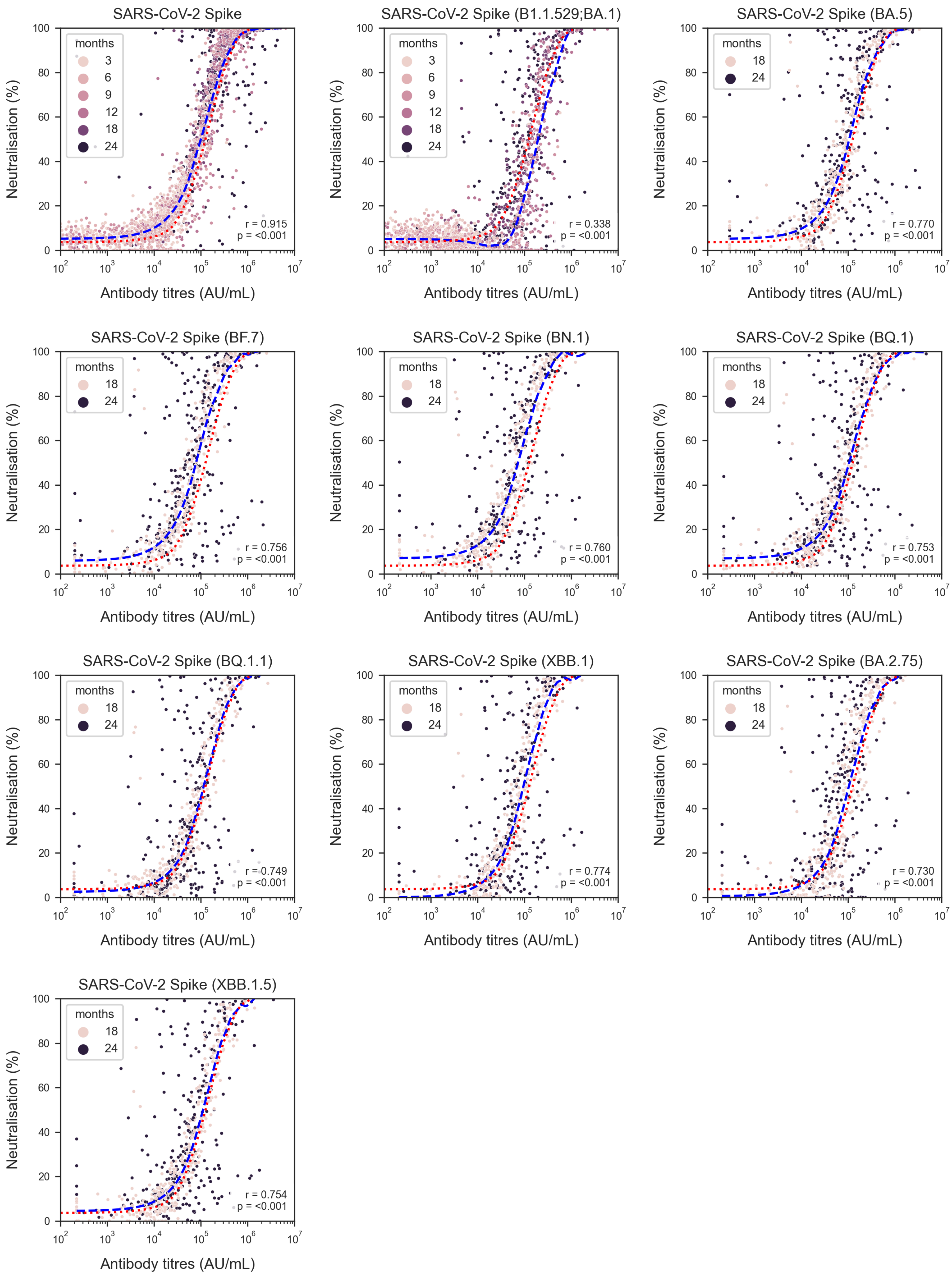

Supplementary Figure S6

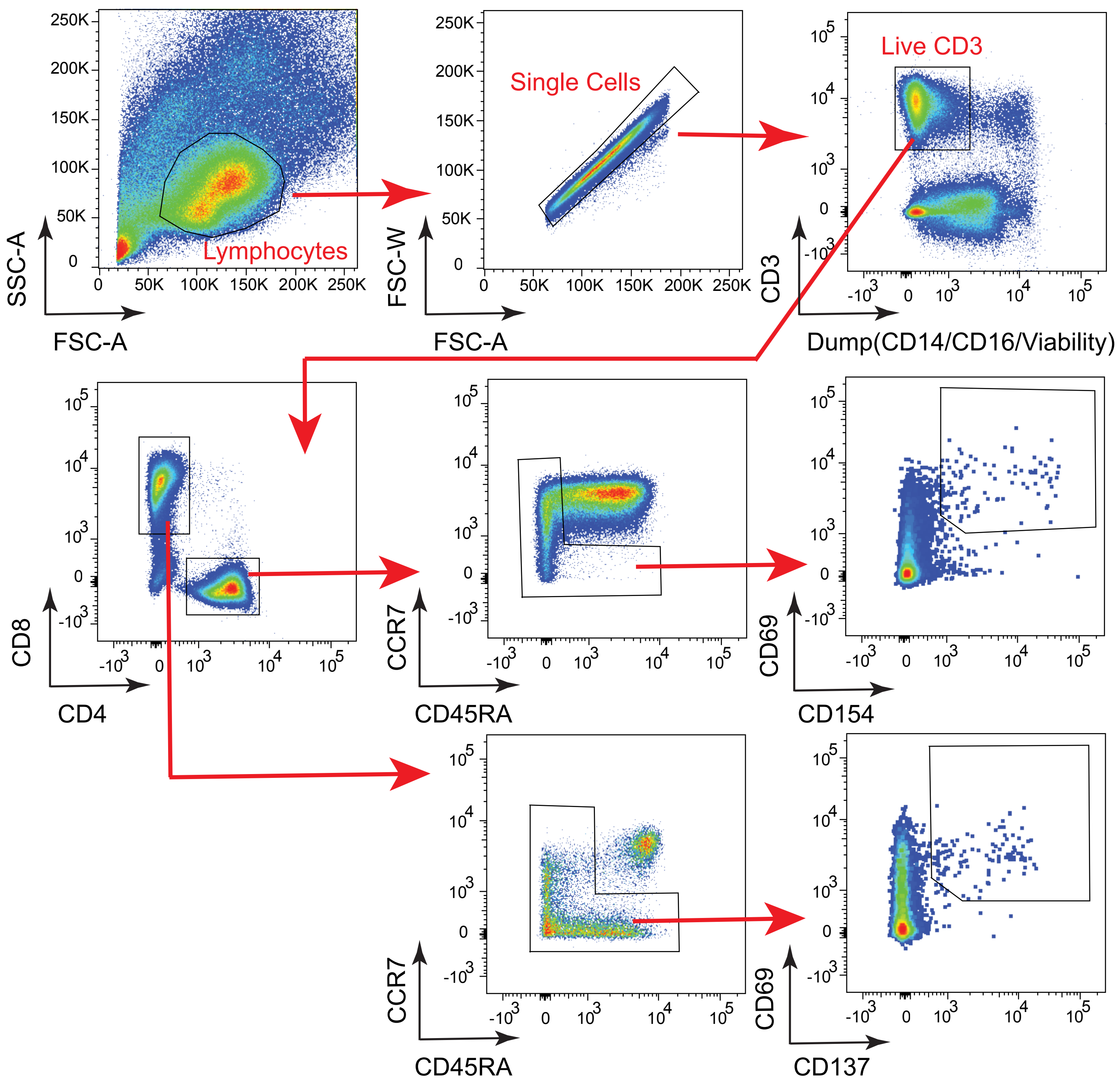

Supplementary Figure S7

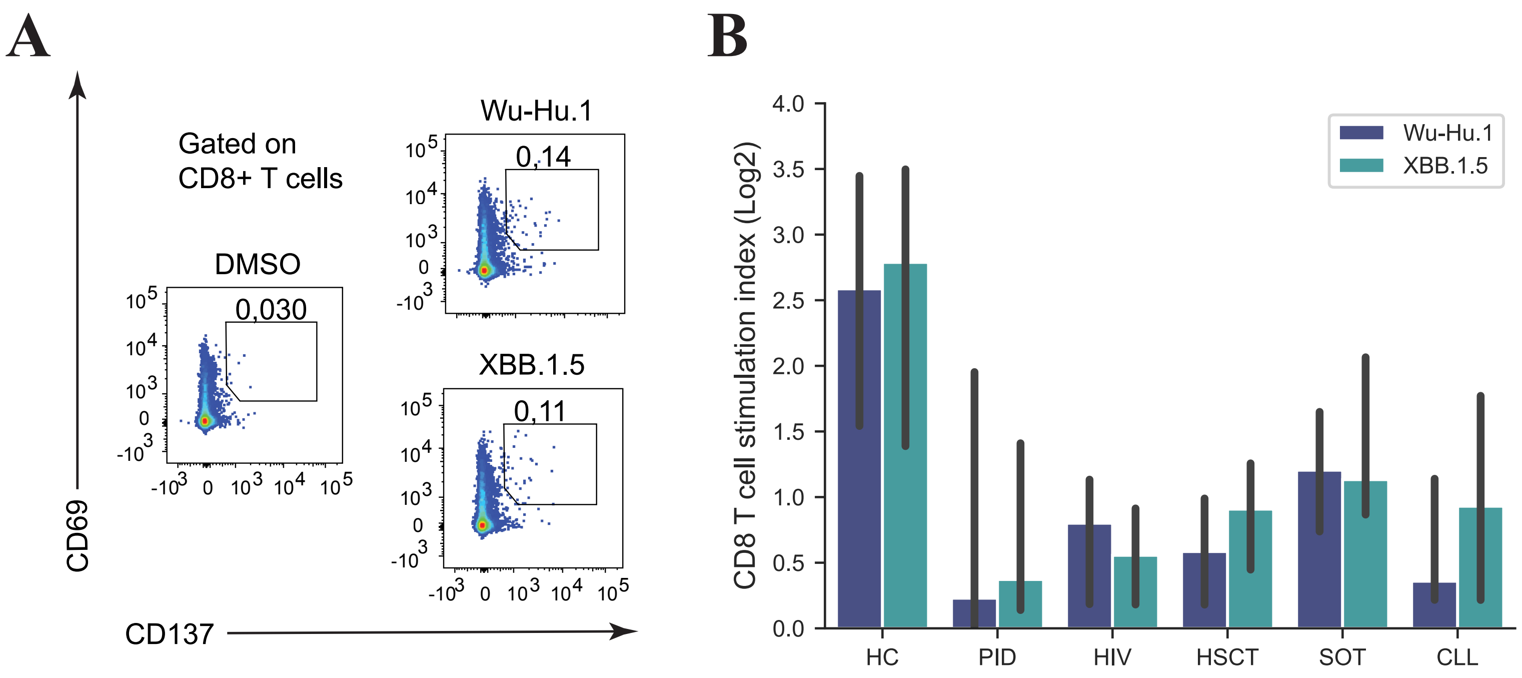

fig	wp	wpsub	comp_1	comp_2	p-value
Figure 1C	HC	N/A	3m	24m	<0.0001
Figure 1C	HC	N/A	6m	24m	<0.0001
Figure 1C	HC	N/A	9m	24m	0.9028
Figure 1C	HC	N/A	12m	24m	0.5611
Figure 1C	HC	N/A	18m	24m	0.2923
Figure 1C	PID	N/A	3m	24m	<0.0001
Figure 1C	PID	N/A	6m	24m	<0.0001
Figure 1C	PID	N/A	9m	24m	<0.0001
Figure 1C	PID	N/A	12m	24m	0.0044
Figure 1C	PID	N/A	18m	24m	0.1146
Figure 1C	HIV	N/A	3m	24m	<0.0001
Figure 1C	HIV	N/A	6m	24m	<0.0001
Figure 1C	HIV	N/A	9m	24m	0.2906
Figure 1C	HIV	N/A	12m	24m	0.3473
Figure 1C	HIV	N/A	18m	24m	0.4874
Figure 1C	HSCT	N/A	3m	24m	<0.0001
Figure 1C	HSCT	N/A	6m	24m	<0.0001
Figure 1C	HSCT	N/A	9m	24m	0.0001
Figure 1C	HSCT	N/A	12m	24m	0.8815
Figure 1C	HSCT	N/A	18m	24m	0.4034
Figure 1C	SOT	N/A	3m	24m	<0.0001
Figure 1C	SOT	N/A	6m	24m	<0.0001
Figure 1C	SOT	N/A	9m	24m	<0.0001
Figure 1C	SOT	N/A	12m	24m	0.5258
Figure 1C	SOT	N/A	18m	24m	0.4381
Figure 1C	CLL	N/A	3m	24m	<0.0001
Figure 1C	CLL	N/A	6m	24m	<0.0001
Figure 1C	CLL	N/A	9m	24m	<0.0001
Figure 1C	CLL	N/A	12m	24m	0.0702
Figure 1C	CLL	N/A	18m	24m	0.5371
Figure 2C	HC	N/A	Spike (Wu-Hu.1)	Spike (B1.1.529;BA.1)	<0.0001
Figure 2C	HC	N/A	Spike (Wu-Hu.1)	Spike (BQ.1)	<0.0001
Figure 2C	HC	N/A	Spike (Wu-Hu.1)	Spike (XBB.1.5)	<0.0001
Figure 2C	PID	N/A	Spike (Wu-Hu.1)	Spike (B1.1.529;BA.1)	<0.0001
Figure 2C	PID	N/A	Spike (Wu-Hu.1)	Spike (BQ.1)	<0.0001
Figure 2C	PID	N/A	Spike (Wu-Hu.1)	Spike (XBB.1.5)	<0.0001
Figure 2C	HIV	N/A	Spike (Wu-Hu.1)	Spike (B1.1.529;BA.1)	<0.0001
Figure 2C	HIV	N/A	Spike (Wu-Hu.1)	Spike (BQ.1)	<0.0001
Figure 2C	HIV	N/A	Spike (Wu-Hu.1)	Spike (XBB.1.5)	<0.0001
Figure 2C	HSCT	N/A	Spike (Wu-Hu.1)	Spike (B1.1.529;BA.1)	<0.0001
Figure 2C	HSCT	N/A	Spike (Wu-Hu.1)	Spike (BQ.1)	<0.0001
Figure 2C	HSCT	N/A	Spike (Wu-Hu.1)	Spike (XBB.1.5)	<0.0001
Figure 2C	SOT	N/A	Spike (Wu-Hu.1)	Spike (B1.1.529;BA.1)	<0.0001
Figure 2C	SOT	N/A	Spike (Wu-Hu.1)	Spike (BQ.1)	0.0012
Figure 2C	SOT	N/A	Spike (Wu-Hu.1)	Spike (XBB.1.5)	<0.0001
Figure 2C	CLL	N/A	Spike (Wu-Hu.1)	Spike (B1.1.529;BA.1)	<0.0001
Figure 2C	CLL	N/A	Spike (Wu-Hu.1)	Spike (BQ.1)	<0.0001
Figure 2C	CLL	N/A	Spike (Wu-Hu.1)	Spike (XBB.1.5)	<0.0001
Supp Figure 2	HC	>60 yrs	3m	24m	<0.0001
Supp Figure 2	HC	>60 yrs	6m	24m	<0.0001
Supp Figure 2	HC	>60 yrs	9m	24m	0.1115
Supp Figure 2	HC	>60 yrs	12m	24m	0.2154
Supp Figure 2	HC	>60 yrs	18m	24m	0.4732
Supp Figure 2	HC	40-59 yrs	3m	24m	<0.0001
Supp Figure 2	HC	40-59 yrs	6m	24m	<0.0001
Supp Figure 2	HC	40-59 yrs	9m	24m	0.4835
Supp Figure 2	HC	40-59 yrs	12m	24m	0.7248
Supp Figure 2	HC	40-59 yrs	18m	24m	0.3432
Supp Figure 2	HC	18-39 yrs	3m	24m	<0.0001
Supp Figure 2	HC	18-39 yrs	6m	24m	<0.0001
Supp Figure 2	HC	18-39 yrs	9m	24m	0.6997
Supp Figure 2	HC	18-39 yrs	12m	24m	0.7870
Supp Figure 2	HC	18-39 yrs	18m	24m	1.0000
Supp Figure 2	PID	CVID	3m	24m	<0.0001
Supp Figure 2	PID	CVID	6m	24m	<0.0001
Supp Figure 2	PID	CVID	9m	24m	<0.0001
Supp Figure 2	PID	CVID	12m	24m	<0.0001
Supp Figure 2	PID	CVID	18m	24m	0.0084
Supp Figure 2	PID	XLA	3m	24m	0.1333
Supp Figure 2	PID	XLA	6m	24m	0.5333
Supp Figure 2	PID	XLA	9m	24m	0.3333
Supp Figure 2	PID	XLA	12m	24m	0.3333

Supp Figure 2	PID	XLA	18m	24m	0.3333
Supp Figure 2	PID	Monogenic disease	3m	24m	0.0411
Supp Figure 2	PID	Monogenic disease	6m	24m	0.0426
Supp Figure 2	PID	Monogenic disease	9m	24m	0.1775
Supp Figure 2	PID	Monogenic disease	12m	24m	1.0000
Supp Figure 2	PID	Monogenic disease	18m	24m	0.5887
Supp Figure 2	PID	CD4-cytopenia	3m	24m	0.0006
Supp Figure 2	PID	CD4-cytopenia	6m	24m	0.0003
Supp Figure 2	PID	CD4-cytopenia	9m	24m	0.2453
Supp Figure 2	PID	CD4-cytopenia	12m	24m	0.7513
Supp Figure 2	PID	CD4-cytopenia	18m	24m	0.5495
Supp Figure 2	PID	Other	3m	24m	0.0154
Supp Figure 2	PID	Other	6m	24m	<0.0001
Supp Figure 2	PID	Other	9m	24m	0.2086
Supp Figure 2	PID	Other	12m	24m	0.2593
Supp Figure 2	PID	Other	18m	24m	0.1649
Supp Figure 2	HIV	≤CD4 300	3m	24m	<0.0001
Supp Figure 2	HIV	≤CD4 300	6m	24m	<0.0001
Supp Figure 2	HIV	≤CD4 300	9m	24m	0.5316
Supp Figure 2	HIV	≤CD4 300	12m	24m	0.7237
Supp Figure 2	HIV	≤CD4 300	18m	24m	0.1749
Supp Figure 2	HIV	>CD4 300	3m	24m	<0.0001
Supp Figure 2	HIV	>CD4 300	6m	24m	<0.0001
Supp Figure 2	HIV	>CD4 300	9m	24m	0.3674
Supp Figure 2	HIV	>CD4 300	12m	24m	0.1934
Supp Figure 2	HIV	>CD4 300	18m	24m	0.9318
Supp Figure 2	HSCT	Early	3m	24m	0.0727
Supp Figure 2	HSCT	Early	6m	24m	0.0242
Supp Figure 2	HSCT	Early	9m	24m	0.4857
Supp Figure 2	HSCT	Early	12m	24m	0.2571
Supp Figure 2	HSCT	Early	18m	24m	0.7619
Supp Figure 2	HSCT	Intermediate	3m	24m	<0.0001
Supp Figure 2	HSCT	Intermediate	6m	24m	<0.0001
Supp Figure 2	HSCT	Intermediate	9m	24m	0.0653
Supp Figure 2	HSCT	Intermediate	12m	24m	0.2766
Supp Figure 2	HSCT	Intermediate	18m	24m	0.0830
Supp Figure 2	HSCT	Late	3m	24m	<0.0001
Supp Figure 2	HSCT	Late	6m	24m	<0.0001
Supp Figure 2	HSCT	Late	9m	24m	0.0007
Supp Figure 2	HSCT	Late	12m	24m	0.6989
Supp Figure 2	HSCT	Late	18m	24m	0.5953
Supp Figure 2	HSCT	CAR-T	3m	24m	0.6667
Supp Figure 2	HSCT	CAR-T	6m	24m	0.5000
Supp Figure 2	HSCT	CAR-T	9m	24m	1.0000
Supp Figure 2	HSCT	CAR-T	12m	24m	1.0000
Supp Figure 2	HSCT	CAR-T	18m	24m	1.0000
Supp Figure 2	SOT	≤6mo w/ MMF	3m	24m	<0.0001
Supp Figure 2	SOT	≤6mo w/ MMF	6m	24m	<0.0001
Supp Figure 2	SOT	≤6mo w/ MMF	9m	24m	0.0034
Supp Figure 2	SOT	≤6mo w/ MMF	12m	24m	0.1292
Supp Figure 2	SOT	≤6mo w/ MMF	18m	24m	0.7434
Supp Figure 2	SOT	>6mo w/ MMF	3m	24m	<0.0001
Supp Figure 2	SOT	>6mo w/ MMF	6m	24m	0.0001
Supp Figure 2	SOT	>6mo w/ MMF	9m	24m	0.0082
Supp Figure 2	SOT	>6mo w/ MMF	12m	24m	0.8918
Supp Figure 2	SOT	>6mo w/ MMF	18m	24m	0.4817
Supp Figure 2	SOT	>6mo w/o MMF	3m	24m	<0.0001
Supp Figure 2	SOT	>6mo w/o MMF	6m	24m	<0.0001
Supp Figure 2	SOT	>6mo w/o MMF	9m	24m	0.0003
Supp Figure 2	SOT	>6mo w/o MMF	12m	24m	0.6268
Supp Figure 2	SOT	>6mo w/o MMF	18m	24m	0.8225
Supp Figure 2	CLL	Ibrutinib	3m	24m	<0.0001
Supp Figure 2	CLL	Ibrutinib	6m	24m	<0.0001
Supp Figure 2	CLL	Ibrutinib	9m	24m	0.0010
Supp Figure 2	CLL	Ibrutinib	12m	24m	0.0158
Supp Figure 2	CLL	Ibrutinib	18m	24m	0.3149
Supp Figure 2	CLL	Off Ibrutinib	3m	24m	0.0650
Supp Figure 2	CLL	Off Ibrutinib	6m	24m	0.0117
Supp Figure 2	CLL	Off Ibrutinib	9m	24m	0.3357
Supp Figure 2	CLL	Off Ibrutinib	12m	24m	0.8785
Supp Figure 2	CLL	Off Ibrutinib	18m	24m	0.4619
Supp Figure 2	CLL	Indolent	3m	24m	<0.0001
Supp Figure 2	CLL	Indolent	6m	24m	<0.0001

Supp Figure 2	CLL	Indolent	9m	24m	0.0222
Supp Figure 2	CLL	Indolent	12m	24m	0.7096
Supp Figure 2	CLL	Indolent	18m	24m	0.5584
Supp Figure 2	CLL	BR/FCR	3m	24m	<0.0001
Supp Figure 2	CLL	BR/FCR	6m	24m	<0.0001
Supp Figure 2	CLL	BR/FCR	9m	24m	<0.0001
Supp Figure 2	CLL	BR/FCR	12m	24m	0.0053
Supp Figure 2	CLL	BR/FCR	18m	24m	0.8035
Supp Figure 4	HC	>60 yrs	Spike	Spike (B1.1.529;BA.1)	<0.0001
Supp Figure 4	HC	>60 yrs	Spike	Spike (BQ.1)	<0.0001
Supp Figure 4	HC	>60 yrs	Spike	Spike (XBB.1.5)	<0.0001
Supp Figure 4	HC	40-59 yrs	Spike	Spike (B1.1.529;BA.1)	<0.0001
Supp Figure 4	HC	40-59 yrs	Spike	Spike (BQ.1)	<0.0001
Supp Figure 4	HC	40-59 yrs	Spike	Spike (XBB.1.5)	<0.0001
Supp Figure 4	HC	18-39 yrs	Spike	Spike (B1.1.529;BA.1)	0.0001
Supp Figure 4	HC	18-39 yrs	Spike	Spike (BQ.1)	0.0012
Supp Figure 4	HC	18-39 yrs	Spike	Spike (XBB.1.5)	0.0006
Supp Figure 4	PID	CVID	Spike	Spike (B1.1.529;BA.1)	<0.0001
Supp Figure 4	PID	CVID	Spike	Spike (BQ.1)	<0.0001
Supp Figure 4	PID	CVID	Spike	Spike (XBB.1.5)	<0.0001
Supp Figure 4	PID	XLA	Spike	Spike (B1.1.529;BA.1)	0.3333
Supp Figure 4	PID	XLA	Spike	Spike (BQ.1)	0.3333
Supp Figure 4	PID	XLA	Spike	Spike (XBB.1.5)	0.3333
Supp Figure 4	PID	Monogenic disease	Spike	Spike (B1.1.529;BA.1)	0.1797
Supp Figure 4	PID	Monogenic disease	Spike	Spike (BQ.1)	0.2403
Supp Figure 4	PID	Monogenic disease	Spike	Spike (XBB.1.5)	0.0931
Supp Figure 4	PID	CD4-cytopenia	Spike	Spike (B1.1.529;BA.1)	0.0047
Supp Figure 4	PID	CD4-cytopenia	Spike	Spike (BQ.1)	0.0086
Supp Figure 4	PID	CD4-cytopenia	Spike	Spike (XBB.1.5)	0.0031
Supp Figure 4	PID	Other	Spike	Spike (B1.1.529;BA.1)	0.0021
Supp Figure 4	PID	Other	Spike	Spike (BQ.1)	0.0070
Supp Figure 4	PID	Other	Spike	Spike (XBB.1.5)	0.0070
Supp Figure 4	HIV	≤CD4 300	Spike	Spike (B1.1.529;BA.1)	0.0007
Supp Figure 4	HIV	≤CD4 300	Spike	Spike (BQ.1)	0.0017
Supp Figure 4	HIV	≤CD4 300	Spike	Spike (XBB.1.5)	0.0007
Supp Figure 4	HIV	>CD4 300	Spike	Spike (B1.1.529;BA.1)	<0.0001
Supp Figure 4	HIV	>CD4 300	Spike	Spike (BQ.1)	<0.0001
Supp Figure 4	HIV	>CD4 300	Spike	Spike (XBB.1.5)	<0.0001
Supp Figure 4	HSCT	Early	Spike	Spike (B1.1.529;BA.1)	0.4857
Supp Figure 4	HSCT	Early	Spike	Spike (BQ.1)	0.8857
Supp Figure 4	HSCT	Early	Spike	Spike (XBB.1.5)	0.6857
Supp Figure 4	HSCT	Intermediate	Spike	Spike (B1.1.529;BA.1)	0.0148
Supp Figure 4	HSCT	Intermediate	Spike	Spike (BQ.1)	0.0281
Supp Figure 4	HSCT	Intermediate	Spike	Spike (XBB.1.5)	0.0281
Supp Figure 4	HSCT	Late	Spike	Spike (B1.1.529;BA.1)	<0.0001
Supp Figure 4	HSCT	Late	Spike	Spike (BQ.1)	<0.0001
Supp Figure 4	HSCT	Late	Spike	Spike (XBB.1.5)	<0.0001
Supp Figure 4	HSCT	CAR-T	Spike	Spike (B1.1.529;BA.1)	1.0000
Supp Figure 4	HSCT	CAR-T	Spike	Spike (BQ.1)	1.0000
Supp Figure 4	HSCT	CAR-T	Spike	Spike (XBB.1.5)	1.0000
Supp Figure 4	SOT	≤6mo w/ MMF	Spike	Spike (B1.1.529;BA.1)	0.0135
Supp Figure 4	SOT	≤6mo w/ MMF	Spike	Spike (BQ.1)	0.1904
Supp Figure 4	SOT	≤6mo w/ MMF	Spike	Spike (XBB.1.5)	0.0326
Supp Figure 4	SOT	>6mo w/ MMF	Spike	Spike (B1.1.529;BA.1)	0.0648
Supp Figure 4	SOT	>6mo w/ MMF	Spike	Spike (BQ.1)	0.0812
Supp Figure 4	SOT	>6mo w/ MMF	Spike	Spike (XBB.1.5)	0.0578
Supp Figure 4	SOT	>6mo w/o MMF	Spike	Spike (B1.1.529;BA.1)	0.0079
Supp Figure 4	SOT	>6mo w/o MMF	Spike	Spike (BQ.1)	0.0056
Supp Figure 4	SOT	>6mo w/o MMF	Spike	Spike (XBB.1.5)	0.0021
Supp Figure 4	CLL	Ibrutinib	Spike	Spike (B1.1.529;BA.1)	0.0157
Supp Figure 4	CLL	Ibrutinib	Spike	Spike (BQ.1)	0.2413
Supp Figure 4	CLL	Ibrutinib	Spike	Spike (XBB.1.5)	0.0258
Supp Figure 4	CLL	Off Ibrutinib	Spike	Spike (B1.1.529;BA.1)	0.0308
Supp Figure 4	CLL	Off Ibrutinib	Spike	Spike (BQ.1)	0.1605
Supp Figure 4	CLL	Off Ibrutinib	Spike	Spike (XBB.1.5)	0.0830
Supp Figure 4	CLL	Indolent	Spike	Spike (B1.1.529;BA.1)	0.0014
Supp Figure 4	CLL	Indolent	Spike	Spike (BQ.1)	0.0108
Supp Figure 4	CLL	Indolent	Spike	Spike (XBB.1.5)	0.0049
Supp Figure 4	CLL	BR/FCR	Spike	Spike (B1.1.529;BA.1)	0.0032
Supp Figure 4	CLL	BR/FCR	Spike	Spike (BQ.1)	0.0055
Supp Figure 4	CLL	BR/FCR	Spike	Spike (XBB.1.5)	0.0016

Stable 1. **Non-dichotomized p-values**.
